# Gonadotropin-releasing hormone and growth hormone act as anti-inflammatory factors improving sensory recovery in female rats with thoracic spinal cord injury

**DOI:** 10.3389/fnins.2023.1164044

**Published:** 2023-06-09

**Authors:** Carlos Guillermo Martínez-Moreno, Denisse Calderón-Vallejo, Carmen Díaz-Galindo, Irma Hernández-Jasso, Juan David Olivares-Hernández, José Ávila-Mendoza, David Epardo, Jerusa Elienai Balderas-Márquez, Valeria Alejandra Urban-Sosa, Rosario Baltazar-Lara, Martha Carranza, Maricela Luna, Carlos Arámburo, José Luis Quintanar

**Affiliations:** ^1^Departamento de Neurobiología Celular y Molecular, Instituto de Neurobiología, Universidad Nacional Autónoma de México, Juriquilla, Querétaro, México; ^2^Departamento de Fisiología y Farmacología, Centro de Ciencias Básicas, Universidad Autónoma de Aguascalientes, Aguascalientes, México

**Keywords:** GH, GnRH, proinflammatory, glial activity markers, sensory recovery, spinal cord injury (SCI), neurotrophic, gene-expression

## Abstract

The potential for novel applications of classical hormones, such as gonadotropin-releasing hormone (GnRH) and growth hormone (GH), to counteract neural harm is based on their demonstrated neurotrophic effects in both *in vitro* and *in vivo* experimental models and a growing number of clinical trials. This study aimed to investigate the effects of chronic administration of GnRH and/or GH on the expression of several proinflammatory and glial activity markers in damaged neural tissues, as well as on sensory recovery, in animals submitted to thoracic spinal cord injury (SCI). Additionally, the effect of a combined GnRH + GH treatment was examined in comparison with single hormone administration. Spinal cord damage was induced by compression using catheter insufflation at thoracic vertebrae 10 (T10), resulting in significant motor and sensory deficits in the hindlimbs. Following SCI, treatments (GnRH, 60 μg/kg/12 h, IM; GH, 150 μg/kg/24 h, SC; the combination of both; or vehicle) were administered during either 3 or 5 weeks, beginning 24 h after injury onset and ending 24 h before sample collection. Our results indicate that a chronic treatment with GH and/or GnRH significantly reduced the expression of proinflammatory (IL6, IL1B, and iNOS) and glial activity (Iba1, CD86, CD206, vimentin, and GFAP) markers in the spinal cord tissue and improved sensory recovery in the lesioned animals. Furthermore, we found that the caudal section of the spinal cord was particularly responsive to GnRH or GH treatment, as well as to their combination. These findings provide evidence of an anti-inflammatory and glial-modulatory effect of GnRH and GH in an experimental model of SCI and suggest that these hormones can modulate the response of microglia, astrocytes, and infiltrated immune cells in the spinal cord tissue following injury.

## 1. Introduction

Spinal cord injury (SCI) is a traumatic event that substantially deteriorates the life quality of patients, and, unfortunately in many cases, only a limited number of therapeutic alternatives are currently available. The spinal tissue disruption that occurs during a mechanical injury leads to an immediate massive loss of neurons and glial cells in the damaged area, followed by the activation of glial cells and an acute inflammatory response involving increased activity of microglia, astrocytes, and infiltrated macrophages/lymphocytes (Freyermuth-Trujillo et al., 2022). The complex interactions between infiltrated immune cells with the resident population of glial cells in the spinal cord commonly induce an exacerbated proinflammatory microenvironment that results in the formation of a glial scar (Okada et al., 2018). Uncontrolled reactive astrogliosis, microglial hyperactivity, and the extracellular deposition of matrix components are responsible for the physical blockade of axonal regeneration (Bellver-Landete et al., 2019; Guerout, 2021). Microglia has a critical role during a neuroinflammatory process, either with or without blood–brain barrier (BBB) disruption, and its response mechanisms under pathophysiological conditions are now the focus of intense research.

The ablation of microglial cells or its hyperactivity has been related to bad prognosis after hurtful events, such as traumatic brain injury (TBI) and SCI, whereas for efficient neurological healing a balanced activation of microglia without acute phenotype polarization into M1 (proinflammatory) or M2 (neurotrophic) is needed (Faden et al., 2016; Fan et al., 2018; Bellver-Landete et al., 2019). It is remarkable that just seconds after the injury, resident microglial cells are activated and simultaneously interact with neurons, astrocytes, infiltrating monocytes, and endothelial cells to generate a complex multi-signaled response directed to protect and regenerate injured neural tissue (Kroner and Rosas Almanza, 2019; Brockie et al., 2021). It has been proposed that interactions between astrocytes, monocytes/macrophages, and microglia and particularly the balance among their polarized phenotypes are fundamental to orchestrate an efficient neuronal restorative process after a lesion (Hu et al., 2015). During this orchestrated cellular response, there are emergency mechanisms that trigger a complex communication system that involves the synthesis, release, and biological action of classical neurotrophins (e.g., brain-derived neurotrophic factor, BDNF; neurotrophin-3, NT3), growth factors (e.g., insulin-like growth factor-1, IGF-1; fibroblast growth factors, FGFs), neurohormones (e.g., growth hormone, GH; gonadotropin-releasing hormone, GnRH), and cytokines (e.g., interleukins, interferons) (Díaz-Galindo et al., 2020). To our knowledge, information about the role of GnRH and GH upon glial activity and their consequential effects on neuroinflammation is scarce.

In recent years, an increasing number of studies regarding the neurotrophic and anti-inflammatory actions of “old” hypothalamic/hypophyseal hormones such as gonadotropin-releasing hormone (GnRH) and growth hormone (GH) have appeared (Bianchi et al., 2017; Martínez-Moreno et al., 2018a; Díaz-Galindo et al., 2020). Furthermore, it has been shown that both GnRH and GH have ubiquitous extrapituitary and extrahypothalamic expression sites, including the central and peripheral nervous system, which implies the existence of fine-tuned autocrine, paracrine, and/or endocrine communication mechanisms in those locations as their corresponding receptors often show a similar distribution (Harvey and Hull, 2003; Millar, 2005; Chu et al., 2013; Arámburo et al., 2014).

GnRH is a hypothalamic peptide that regulates the pituitary–gonad axis canonical reproductive actions in all vertebrate species (Spaziani et al., 2021). Recently, new neurotrophic roles associated with this peptide, particularly in the SCI model, have been reported, and the pervasive distribution of its receptor within the CNS suggests specific actions outside the reproductive physiology. Moreover, chronic administration of GnRH or leuprolide acetate (LA)—a GnRH analog—for 5 weeks consistently improved urodynamic parameters and sensorimotor function in rats with SCI (Calderón-Vallejo and Quintanar, 2012; Calderón-Vallejo et al., 2015; Díaz Galindo et al., 2015). In humans, the presence of GnRH receptor (GnRHR) in spinal cord tissue has been confirmed and it is proposed that some of the beneficial actions observed in SCI rats and patients treated with LA are, at least, partially mediated through local GnRHR activation (Quintanar et al., 2018; Altamira-Camacho et al., 2020; Díaz-Galindo et al., 2021).

Aside from its critical developmental role, GH has now well-accepted actions as a neuroprotective/regenerative factor in several neural harm models (Alba-Betancourt et al., 2013; Ávila-Mendoza et al., 2016; Sanchez-Bezanilla et al., 2020a,b; Baltazar-Lara et al., 2021), which include an increasing number of medical cases and preclinical and clinical trials (Bianchi et al., 2017). Neurotrophic effects of GH have been documented in different areas of the nervous system that include sensorimotor cortex (pallium), hippocampus, cerebellum, retina, spinal cord, and peripheral nerves (Devesa et al., 2012; Olivares-Hernández et al., 2021, 2022; Juárez-Aguilar et al., 2022). Also, GH receptors (GHR) are widely distributed in neurons and glial cells in many species, and its expression is upregulated as a cell emergency mechanism after a neural injury, as reported in neuroretina cells under excitotoxic conditions (Fleming et al., 2018; Martínez-Moreno et al., 2019). Interestingly, it has been shown that traumatic injuries in spinal cord and brain are followed by growth hormone deficiency (GHD) and post-traumatic hypopituitarism (PTHP) associated with long-term poor medical outcomes (Kgosidialwa et al., 2019). GH replacement therapy for GHD in SCI has a low risk of neurological side effects, and when combined with physical rehabilitation, it can improve quality of life; although more research is needed to understand the molecular mechanisms underlying this effect (Cuatrecasas et al., 2018). Recent evidence suggests that GH can also act as an immunomodulator with beneficial effects during immune responses and inflammatory pathologies such as irritable bowel disease (Meazza et al., 2004; Soendergaard et al., 2017).

Here, we aimed to investigate whether the reported neurotrophic actions of GnRH and GH are likely to be associated with an anti-inflammatory effect in the damaged spinal cord. Thus, in this work, we examined gene-expression changes of several proinflammatory and reactive gliosis markers after a mechanical injury in the rat spinal cord at T10 level. We studied three sections of the spinal cord to assess the effects of GnRH and/or GH upon the spinal cord inflammatory response in the epicenter of the lesion and the upper and lower spinal surrounding tissues. Several changes in cell distribution in the spinal cord tissue were observed using specific antibodies against glial molecular markers. Our results showed a positive correlation between molecular and cellular findings with the recovery outcome obtained in the hot-plate sensory test, implying that GnRH and GH exert anti-inflammatory and protective actions in the SCI model.

## 2. Methodology

### 2.1. Animals

Female *Wistar* rats of about 250 g body weight were used for these studies. The animals, produced and reared at the Institute of Neurobiology-UNAM, were acclimatized to a 12-h light and 12-h dark cycle under controlled room temperature (20–22°C) during the experiments. Purina chow pellets and tap water were provided *ad libitum*. All experimental protocols were conducted according to the bioethical guidelines established by the National Health and Medical Research Council and were approved by the corresponding animal ethics committees of the UAA and INb-UNAM. The rats were treated according to the Institutional Regulations on animal welfare (UAA).

### 2.2. Experimental design

**Figure 1:**
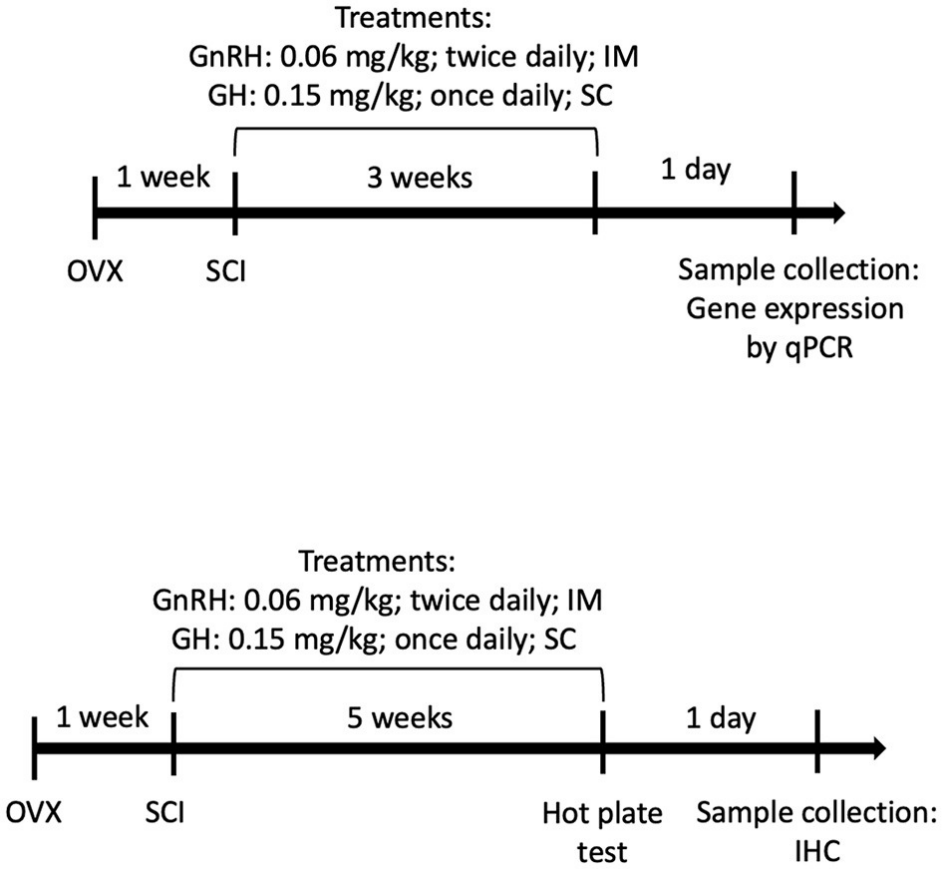
Experimental design timelines: **(Top)** molecular analysis (3 weeks) and **(Bottom)** sensorial/histological analysis (5 weeks).

Using G^*^Power 3.1 software, we obtained a size effect of d = 0.55 (moderate to large), allowing a type error 1 of 5%, alpha: 0.05, with the power of 80% (β = 0.2) for five experimental groups and eight animals per group; in addition, four animals per group were included to cover for dropouts due to injury-related death or bioethical euthanasia. Animals were randomly split into five groups: (1) sham SCI surgery (sham group), (2) spinal cord injury (SCI) treated with physiological saline solution (SCI group), (3) SCI treated with GnRH (GnRH group), (4) SCI treated with GH (GH group), and (5) SCI treated with the combination of both GnRH and GH (G+G group). In the sham group, surgery was performed as usual, but omitting the damaging step: the catheter insufflation. Treatments were administered as follows: Human recombinant gonadotropin-releasing hormone (GnRH, 0.06 mg/kg) (L7134, Sigma, MO, USA) was injected every 12 h by intramuscular (IM) administration (Calderón-Vallejo and Quintanar, 2012); a single daily dose (0.15 mg/kg; Heredia et al., 2021) of human recombinant growth hormone (rhGH) (somatropin; Genotropin C, Pfizer, México) was subcutaneously (SC) administered in the morning; and vehicle (100 μL of saline solution, 0.9% NaCl in distilled water) was administered IM (Figure 1). The treatments started 24 h after the SCI surgery and stopped 24 h before euthanasia. For gene expression quantification analysis, a 3-week chronic administration protocol was followed as it has been reported that astrocyte and microglial/macrophage activity peaks in SCI tissues between 2 and 8 weeks after damage (Kroner and Rosas Almanza, 2019; Freyermuth-Trujillo et al., 2022); for instance, spinal tissues were collected and stored at −70 °C until processing for molecular examination. Following previous studies from our group (Calderón-Vallejo et al., 2015; Díaz Galindo et al., 2015; Díaz-Galindo et al., 2020), an independent set of experiments was performed with a longer treatment protocol (5 weeks) to assess sensory recovery through the hot-plate test and to determine neuroinflammatory cellular activity changes in the injured site and contiguous SCI tissues by immunohistochemical analysis.

### 2.3. Spinal cord injury surgery and postoperative care

Ovariectomized rats were used to avoid GnRH-induced estrogen secretion, as it has been reported that estrogen and progesterone may act as neuroprotectors and thus could interfere and mask the results of this study (Calderón-Vallejo et al., 2015; Díaz Galindo et al., 2015). For ovariectomy (OVX), the rats were anesthetized and the ovaries were surgically removed as previously described (Calderón-Vallejo et al., 2015). Following recovery (1 week after OVX), rats were anesthetized with isoflurane and prepared for spinal cord surgery. SCI was induced by compression with a balloon (Vanicky et al., 2001) with minor modifications as reported before (Calderón-Vallejo and Quintanar, 2012; Díaz Galindo et al., 2015). Briefly, a 2-French Fogarty catheter was inserted into the dorsal epidural space through a small hole made with a surgical drill at the level of the T11 vertebra. The SCI was induced by balloon inflation at the end of the catheter with a volume of 20 μl at the T10 spinal level. The catheter inflation was performed for 5 min to produce posterior paraplegia by mechanical damage in spinal cord tissue. Animals showing complete paraplegia were included in the study, whereas those that showed any movement in their hindlimbs were excluded. After surgery, all the experimental groups were injected with penicillin (Penprocilin; 5,000 IU; IM) once daily for 7 days to prevent infections and with metamizole sodium (Neo-melubrin 15 mg/kg; IM) once daily for 3 days to minimize pain. After SCI surgery, urinary bladders were manually and very carefully drained twice a day or until the bladder reflex was restored. Animals showing extensive stress, pain, or infection were euthanized according to bioethical guidelines.

### 2.4. Gene-expression quantification by real-time polymerase chain reaction

At day 21 postinjury, animals were perfused with saline solution (0.9% NaCl in distilled water) under pentobarbital anesthesia and then quickly euthanized; sample tissues were collected, fast frozen on dry ice, and stored at −70°C until used. Total RNA was purified from spinal cord tissue lysates using the Zymo Direct-zol purification kit and TRIzol (Zymo Research Corp., Irvine, CA, USA). Following RNA extraction using columns for specific binding, DNase I (Promega) digestion was performed at 37°C for 20 min then the enzyme was inactivated at 72°C for 15 min. Complementary DNA (cDNA) was synthesized from 1 μg of total RNA using a combination of oligo (dT) and random hexamers. Retrotranscription (RT) was performed with 100 U of Moloney murine leukemia virus reverse transcriptase (Promega) and 1 mM deoxyribonucleoside triphosphate mix for 60 min at 42°C. Target gene expressions (Table 1) were quantified by real-time quantitative PCR (qPCR) in a sequence detection system QuantStudio (Applied Biosystems, Foster, CA, USA) and using a reaction mix with SYBR Green (Maxima; Thermo Fisher Scientific, Waltham, MA, USA) in 10 μL final volume, containing 3 μL of diluted cDNA (1:3) and 0.5 μM of each primer (forward and reverse). Primers were designed including an intron inside the amplification frame to avoid genomic DNA contamination and efficiency was confirmed with standard curves in cDNA for spinal tissue (Table 1). Reactions were performed under the following conditions: initial denaturation at 95°C for 10 min, followed by 45 cycles of 95°C for 15 s, 60°C for 15 s, and 72°C for 15 s. Dissociation curves were included after each qPCR run to ensure primer specificity. The relative abundance of mRNA was calculated using the comparative threshold cycle (Ct) method and employing the formula 2^−Δ*ΔCT*^ where the quantification is expressed relative to the ribosomal protein S18 (rps18) mRNA.

**Table 1:** Oligonucleotides.

**Target gene**	**Primer**	**Sequence**	**Size**	**Accession #**
IL1b	Fwd	CACCTCTCAAGCAGAGCACAG	79 bp	NM_031512.2
	Rev	GGGTTCCATGGTGAAGTCAAC		
IL6	Fwd	TCCTACCCCAACTTCCAATGCTC	79 bp	NM_012589.2
	Rev	TTGGATGGTCTTGGTCCTTAGCC		
iNOS	Fwd	CCTTGTTCAGCTACGCCTTC	179 bp	NM_012611.3
	Rev	GGTATGCCCGAGTTCTTTCA		
Iba1	Fwd	GCCTCATCGTCATCTCCCCA	142 bp	NM_017196.3
	Rev	AGGAAGTGCTTGTTGATCCCA		
CD86	Fwd	TCAATAGCACTGCATACCTGCC	78 bp	NM_020081.1
	Rev	GCCAAAATACTACGAGCTCACT		
CD206	Fwd	ACTGCGTGGTGATGAAAGG	67 bp	NM_001106123.2
	Rev	TAACCCAGTGGTTGCTCACA		
Vimentin	Fwd	CATGCGGCTGCGAGAAAAAT	113 bp	NM_031140.1
	Rev	GGTCAAGACGTGCCAGAGAA		
GFAP	Fwd	CAGACTTTCTCCAACCTCCAG	138 bp	NM_017009.2
	Rev	CTCCTGCTTCGACTCCTTAATG		
GH	Fwd	GGCCCAGCAGAGAACTGACAT	174 bp	NM_001034848.2
	Rev	ATCAGAGCCTGGATGCCCTC		
GHR	Fwd	ATCTTTGGCGGGTGTTCTTA	78 bp	NM_017094.2
	Rev	TAGCTGGTGTAGCCCCACTT		
GnRHR	Fwd	ATGCAGACGCCATCTTCACC	106	bp NM_031577.1
	Rev	TGGTTCCTCTCCCCTTGCT		
RPS18	Fwd	TTCAGCACATCCTGCGAGTA	136 bp	NM_213557.1
	Rev	TTGGTGAGGTCAATGTCTGC		

### 2.5. Immunohistochemistry and fluorescence microscopy

Animals were perfused with saline solution and then euthanized 5 weeks after SCI. Cervical and lumbar dissections were performed to expose the vertebrae canal that contained the nervous tissue on both sides of the spinal cord (rostral and caudal). Using a 50-ml syringe and a metal adaptor, a strong flow of water was flash pumped into the lumbar vertebrae canal to fully eject the spinal cord from the cervical end in one shot. Spinal cord tissue fixation was performed in 4% paraformaldehyde for 7 days at 4°C, then transferred to 30% sucrose solution for cryoprotection and stored at −20°C until processed for analysis. Three sections of 1.5 cm each were obtained taking the injury puncture (well-defined spot) as the reference center. Spinal cord sections were labeled as follows: (a) cephalic section (proximal, T7–T8), (b) injury site (epicenter, T9–T10), and (c) caudal section (distal, T11–T12). After fixation, tissues were freeze mounted onto aluminum sectioning blocks with Tissue-Tek O.C.T. (Sakura Finetek, Torrance, CA, USA). Sections of 15 μm were cut with a cryostat (Leica CM3050 S, Buffalo Grove, IL, USA) and then mounted on glass slides treated with silane to enhance tissue slice binding. Images were captured using an Olympus BX51 fluorescence microscope (Tokyo, Japan) and analyzed with Image Pro software (Media Cybernetics, Rockville, MD, USA). For immunohistochemical analysis, the cellular distributions of Iba1, myelin basic protein (MBP), and glial fibrillary acidic protein (GFAP) were determined with primary polyclonal antibodies (Table 2) diluted in PBS plus 0.2 % Triton X-100 and 1 % fat-free milk (Bio-Rad). Secondary antibodies (Table 2) were diluted 1:200 in TPBS with 1% milk (Bio-Rad) and incubated for 2 h at RT. Controls without primary antibodies were included. Sections were also counterstained with DAPI, as previously described (Martínez-Moreno et al., 2016).

**Table 2:** Antibodies.

**Target**	**Host/Type**	**Dilution**	**Source**	**Cat.No**
Iba-1	Goat/polyclonal	1:400	Abcam	Ab5076
MBP	Rabbit/polyclonal	1:500	Invitrogen	PA5-78397
GFAP	Rabbit/polyclonal	1:200	Abcam	16997-1
Goat IgG	Rabbit/Alexa fluor 488	1:1000	Invitrogen	A-11078
Rabbit IgG	Goat/Alexa fluor 594	1:1000	Invitrogen	A-11012

### 2.6. Hot-plate test for sensory recovery

The sensory recovery test was performed 5 weeks after the SCI, using a Hot/Cold Plate (Harvard Apparatus) controlled with BSRamp Software, following the procedure previously described by Yalcin et al. (2009). The plate increased by 2°C per second, starting at 48°C and up to 55°C as the top limit temperature. The recording pedal was activated manually to register movement latency and stop the temperature increase once animals lift their hinds from the plate. A 60-s limit was established to avoid harmful exposure to high temperatures.

### 2.7. Statistical analysis

In all the experiments, values are expressed as mean ± SEM. Each dot represents an observation (animal) and significant differences between groups or treatments were determined by one-way ANOVA with Fisher's LSD as *post hoc* test. Asterisks (^*^) show differences compared with control (sham) and pound sign (#) show multiple comparisons among groups. Outliers were ruled out with the ROUT method (Q=1%) using Prism Graph 9 (GraphPad, San Diego, CA, USA). Data were collected from three independent experiments without pseudoreplication.

## 3. Results

### 3.1. Effect of GnRH and GH upon IL1B, IL6, and iNOS after SCI

**Figure 2:**
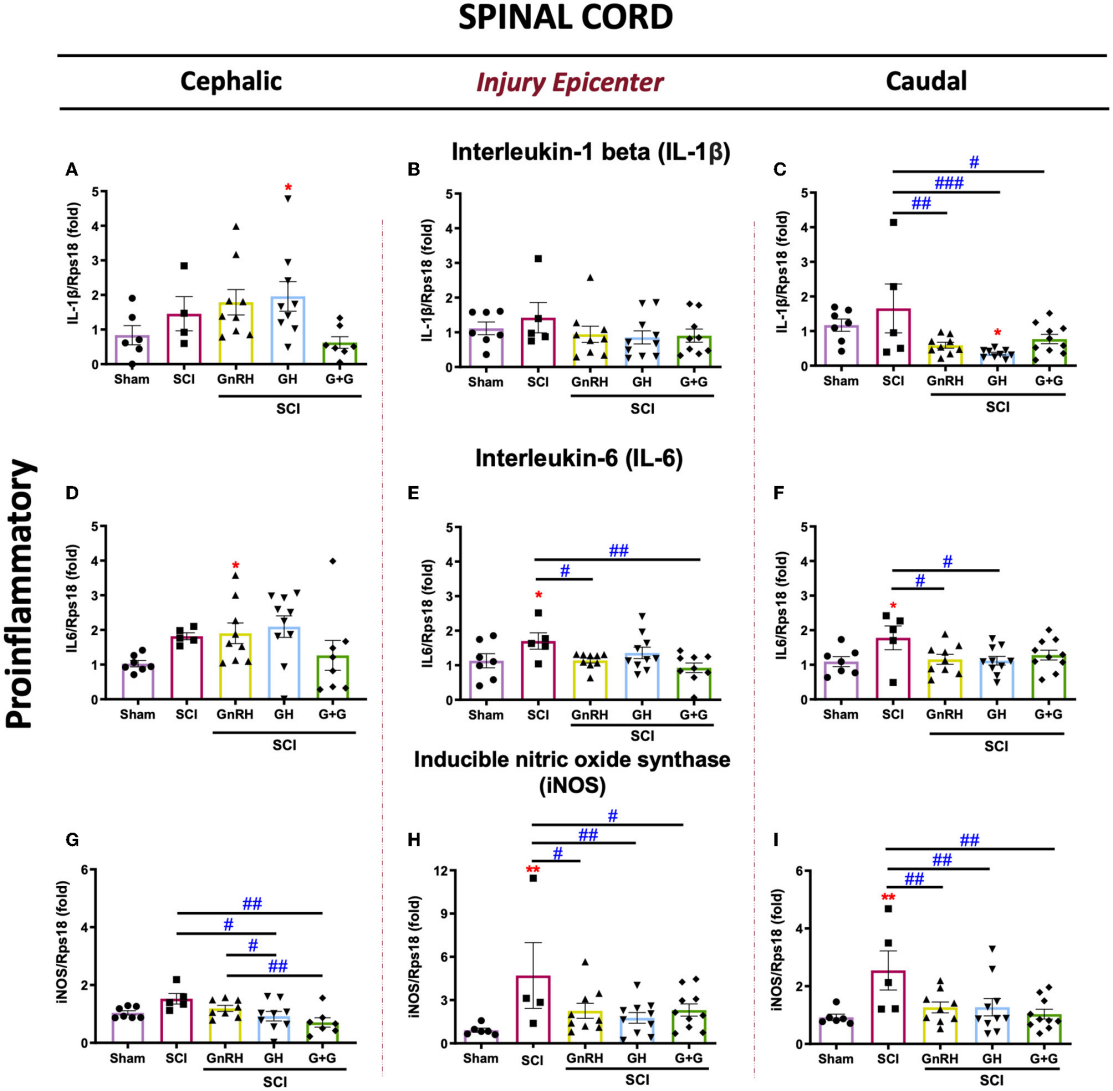
Relative gene expression of IL-1β, IL6, and iNOS (proinflammatory cytokines) in the lesioned spinal cord tissue. **(A–C)** relative change of IL-1β mRNA; **(D, E)** relative change of IL6 mRNA; **(G–I)** relative change of iNOS mRNA. Spinal cord section analyzed (relative to T10): **(A, D, G)** cephalic (proximal)—T9–T8; **(B, E, H)** injury site (damage epicenter)—T10–T11; **(C, F, I)** caudal (distal)—T12–T13. Experimental groups: sham (control), spinal cord injury (SCI), GnRH, GH, and GnRH+GH (G+G). Rps18: reference gene. Results are shown as mean ± SEM, each dot represents an observation (animal); asterisks show differences compared with control (sham) and # show multiple comparisons among groups. ^*, #^*p* < 0.05; ^**, *##*^*p* < 0.01; ^###^*p* < 0.001 determined by one-way ANOVA with LSD Fisher as *post hoc* test.

We analyzed the effect of a chronic (3 weeks) treatment with GH and/or GnRH upon the mRNA expression of several proinflammatory markers (IL1B, IL6, and iNOS, Figure 2) in the lesioned spinal tissue collected from three sections: cephalic spinal cord section (T7–T8), injury section (T9–T10), and distal spinal cord section (T11–T12). In comparison with the SCI group, we found a strong suppressive effect of GH (*p* < 0.001), GnRH (*p* < 0.01), and their combination (G+G; *p* < 0.05) upon IL1β mRNA expression in the caudal section of the spinal cord (Figure 2C). In contrast, no significant changes in this marker were observed in the injury section in any experimental group (Figure 2B), whereas a significant increase (*p* < 0.05) in the GH group was observed in the cephalic section (Figure 2A) in relation to the sham control. The analysis of IL6 expression revealed that SCI induced a significant increase (*p* < 0.05) in the injury site (Figure 2E) and the caudal spinal cord section (Figure 2F). However, GnRH administration reduced substantially the transcription of IL6 mRNA that was induced by the insult in both injured (*p* < 0.05) and caudal (*p* < 0.05) sections (Figures 2E, F, respectively). Furthermore, in the injury site, the combination of hormones (G+G group) significantly reduced (*p* < 0.01) the levels of IL6 mRNA in comparison with the SCI group, whereas GH treatment restored IL6 transcription levels to sham expression rate, although no statistical difference was observed in relation to SCI group (Figure 2E). Individual treatments of GH or GnRH induced a significant reduction (*p* < 0.05) in IL6 transcription level in the caudal section of the spinal cord, but their combination only returned its expression to sham levels without a significant difference when it was compared with the SCI group (Figure 2F). Finally, results showed that the expression levels of iNOS mRNA were significantly increased (*p* < 0.01) in the SCI group, in both the injury epicenter (Figure 2H) and the caudal SC section (Figure 2I); interestingly, in both areas, this upregulation was strongly suppressed by GnRH (*p* < 0.05 and *p* < 0.01), GH (*p* < 0.01), or their combination (*p* < 0.05 and *p* < 0.01), respectively. Also, in the cephalic SC section, GH administration alone (P <0.05) and combined with GnRH (*p* < 0.019) significantly decreased the iNOS mRNA expression in comparison with SCI group (Figure 2G). In contrast, GnRH did not exert any effect upon the expression of iNOS in the cephalic section of the SCI model. It should be noticed that in the caudal section of the spinal cord collected 3 weeks after the injury, the treatments with either GH or GnRH clearly reduced the expression of all the proinflammatory markers determined in this study.

### 3.2. Effect of GnRH and GH upon glial activity after SCI

**Figure 3:**
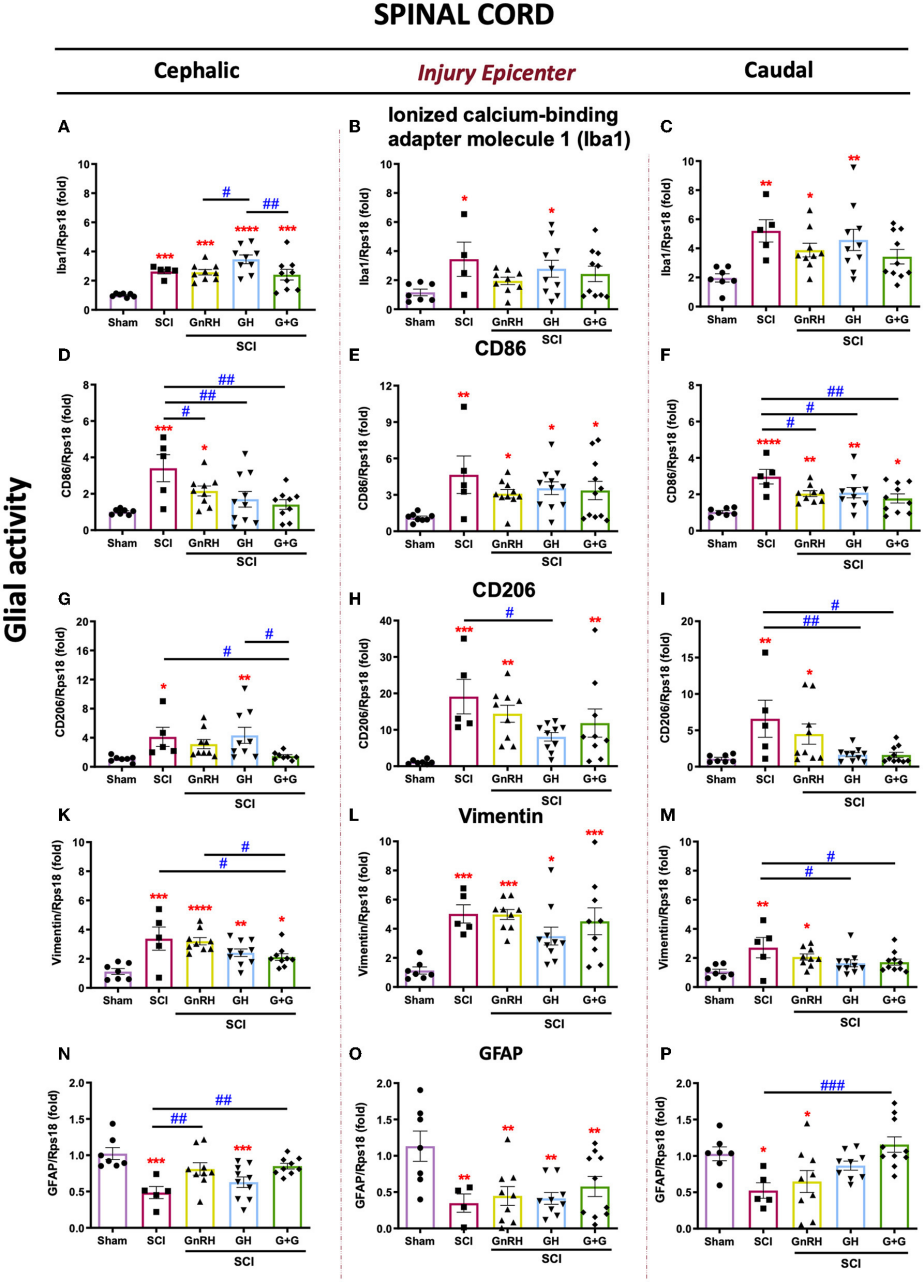
Relative gene expression of Iba1, CD86, CD206, vimentin, and GFAP (glial activity markers) in the lesioned spinal cord tissue. **(A–C)** relative change of Iba1 mRNA; **(D, F)** relative change of CD86 mRNA; **(G–I)** relative change of CD206 mRNA; **(K–M)** relative change of vimentin mRNA; **(N–P)** relative change of GFAP mRNA. Spinal cord section analyzed (relative to T10): **(A, D, G, K, N)** cephalic (proximal)—T9–T8; **(B, E, H, L, O)** injury site (damage epicenter)—T10–T11, and **(C, F, I, M, P)** caudal (distal)—T12–T13. Experimental groups: sham (control), spinal cord injury (SCI), GnRH, GH, and GnRH+GH (G+G). Rps18: reference gene. Results are shown as mean ± SEM, each dot represents an observation (animal); asterisks show differences compared with control (sham) and # show multiple comparisons among groups ^*, #^*p* < 0.05; ^**, *##*^*p* < 0.01; ***p < 0.001; ****P < 0.0001 determined by one-way ANOVA with LSD Fisher as *post hoc* test.

The expression of Iba1, CD86, CD206, vimentin, and GFAP mRNAs was analyzed as molecular markers for glial activity in the SCI model. In the nervous system, Iba1 is widely used as a cell marker for macrophage infiltration and microglia. As shown in Figure 3A, Iba1 expression significantly changed in all groups in the rostral SC tissue in comparison with the sham control. Conversely, in the SCI group, Iba1 notably increased its transcription rate in both the lesion site (Figure 3B, *p* < 0.05) and the caudal spinal cord section (Figure 3C, *p* < 0.01), respectively, in comparison with the sham control. No significant changes were observed with the treatments in the injury epicenter and distal section (Figures 3B, C); however, the combined treatment (G+G) reverted the SCI-induced upregulation in both regions, as also did GnRH in the injured area. However, results showed that SCI significantly increased the proinflammatory phenotype marker CD86 in the three sections that were analyzed in comparison with sham-control group (Figures 3D–F). In the rostral section, all the treatments (GnRH, *p* < 0.05; GH and G+G, <0.01; respectively) provoked a clear reduction in the levels of CD86 mRNA in comparison with the SCI group (Figure 3D). In the lesioned area, SCI induced a strong stimulation (*p* < 0.01) of CD86 mRNA transcription but showed a reduction in its *p*-value from 0.01 to 0.05 in animals treated with GH, GnRH, or their combination (Figure 3E). As mentioned above, the caudal section of SC showed a significant increase in CD86 mRNA induced by SCI, but this was significantly reduced (*p* < 0.05) in both the GH or GnRH groups and the group treated with the combination of hormones (*p* < 0.01) (Figure 3F). It was also found that SCI induced a strong increase in the transcriptional rate of CD206 (also known as MMR, a mannose-receptor used as a marker for M2 macrophage/microglia phenotype) in the three regions; although this upregulation was exacerbated (around a 20-fold rise) in the injury epicenter. In the cephalic spinal tissue, the individual treatments did not exert significant effects in comparison with the SCI group, but G+G treatment reduced (*p* < 0.01) its expression. However, it was found that in the lesion site GH significantly reduced CD206 expression (*p* < 0.05) in relation to the injured group. Additionally, a decrease in *p*-value from 0.001 to 0.01 was observed in the GnRH and G+G groups, when compared with the sham group, although no statistical difference was found in relation to the SCI group. Results also showed that, in the distal SC section, both GH and G+G treatments significantly reduced CD206 mRNA expression (*p* < 0.01 and *p* < 0.05, respectively), while GnRH administration only lowered the *p*-value in comparison with that observed in the SCI group. Vimentin is an accepted marker for glial activation as the astrocytes/microglia increase their mRNA expression after an injury in neural tissue. Here, results showed that vimentin was increased in the three SCI studied sections (Figures 3K–M). After the lesion, vimentin mRNA expression increased 5-fold (*p* < 0.001) in the injury epicenter site, whereas it augmented 3.5-fold in the rostral tissue (*p* < 0.001) (Figures 3K, L). No statistically significant changes were observed with the treatments in the injury region in comparison with the SCI group. However, in the cephalic tissue, vimentin mRNA expression was significantly reduced (*p* < 0.05) with the combined treatment (Figure 3K). Also, in the caudal section SCI significantly increased vimentin expression, whereas both GH and G+G treatments completely blocked this upregulation; instead, no difference was observed between GnRH and SCI groups despite *p*-value going down from 0.01 to 0.05 (Figure 3M). Finally, GFAP expression was studied to assess glial activity after SCI (Figures 3N–P). Our results showed that GFAP mRNA levels were significantly downregulated in the SCI group in all three sections analyzed. In the cephalic portion of the spinal cord, both the GnRH and G+G groups were able to revert the injury-induced reduction in GFAP mRNA expression (*p* < 0.01) (Figure 3N). In the distal section, only the G+G treatment was able to significantly recuperate mRNA levels to those found in the sham control (*p* < 0.001) (Figure 3P); in contrast, in the injury epicenter, no treatment was able to restore levels (Figure 3O).

### 3.3. Effect of GnRH and GH upon GH, GHR, and GnRHR gene expression after SCI

**Figure 4:**
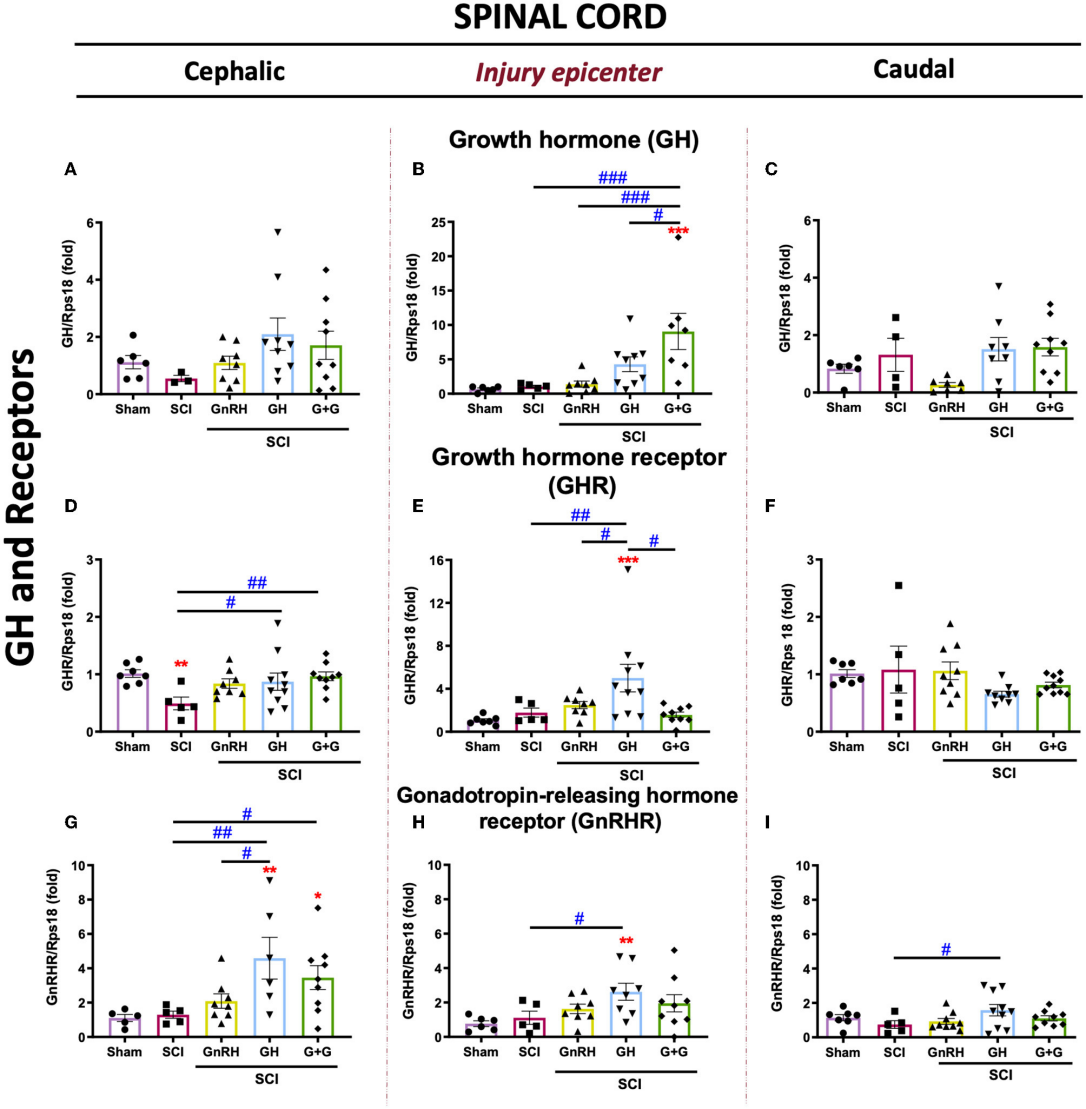
Relative gene expression of GH, GHR, and GnRHR in the lesioned spinal cord tissue. **(A–C)** relative change of GH mRNA; **(D, E)** relative change of GHR mRNA, and **(G–I)** relative change of GnRHR mRNA. Spinal cord section analyzed (relative to T10): **(A, D, G)** cephalic (proximal)—T9–T8; **(B, E, H)** injury site (damage epicenter)—T10–T11 and **(C, F, I)** caudal (distal)—T12–T13. Experimental groups: sham (control), spinal cord injury (SCI), GnRH, GH, and GnRH+GH (G+G). Rps18: reference gene. Results are shown as mean ± SEM, each dot represents an observation (animal); asterisks show differences compared with control (sham), and # show multiple comparisons among groups ^*, #^*p* < 0.05; ^**, *##*^*p* < 0.01; ***p < 0.001 determined by one-way ANOVA with LSD Fisher as *post hoc* test.

The expression of GH, GHR, and GnRHR mRNAs in injured animals treated with GH, GnRH, and G+G (combined treatment) is shown in Figure 4. Although there was a tendency to increase locally expressed GH, no statistically significant changes were found in any experimental condition in the cephalic nor caudal SC portions (Figures 4A, C). In the epicenter, however, the G+G treatment induced a higher GH mRNA expression (Figure 4B). However, GHR mRNA expression was importantly depleted after the compressive lesion in the rostral section, and both GH (*p* < 0.05) and G+G (*p* < 0.01) treatments restored its levels to those similar in the sham control (Figure 4D). Curiously, GHR mRNA did not change in the injury and caudal SC portions in the untreated damaged animals (Figures 4D–F). Interestingly, GH induced a strong upregulation of GnRHR mRNA in comparison with the SCI group in both rostral (4-fold) and injured (2.5-fold) tissues, whereas in the caudal section, it also showed a significant increase (*p* < 0.05). Intriguingly, in the injury epicenter, the combined treatment (GnRH+GH) provoked that the significant difference observed in the GH group (*p* < 0.01) was abolished in the G+G group (Figures 4G–I).

### 3.4. Effect of GnRH and GH upon GFAP-immunoreactivity after SCI

**Figure 5:**
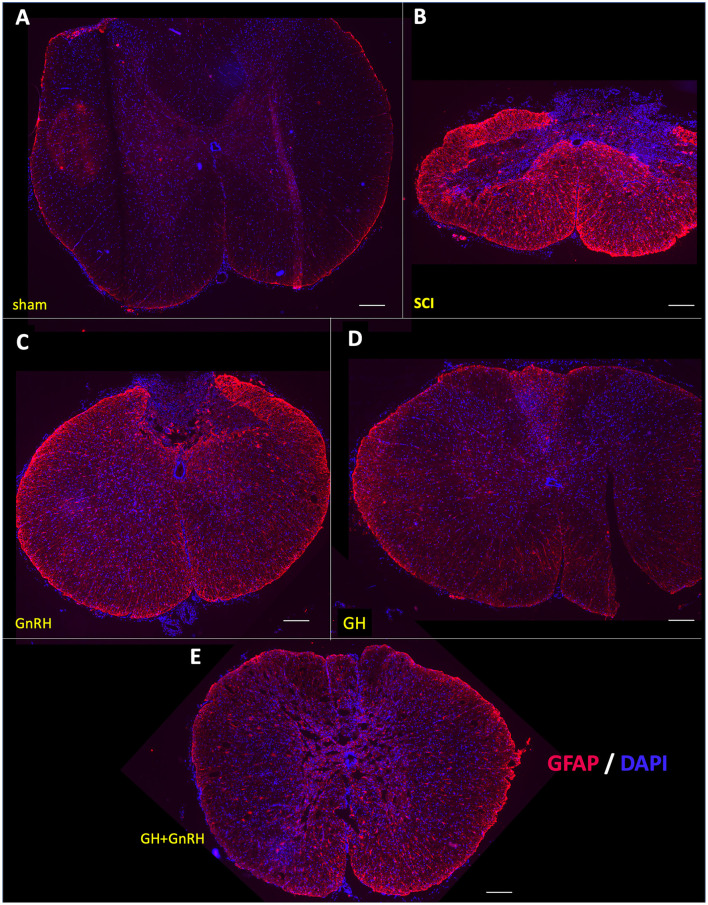
Glial fibrillary acidic protein (GFAP) immunohistochemistry in spinal cord tissue. Transverse sections of the spinal cord collected from the distal side of the injury (T11–T12) at 4x magnification. Experimental groups: **(A)** sham (control), **(B)** spinal cord injury without treatment (SCI), **(C)** GnRH, **(D)** GH, and **(E)** GnRH+GH (G+G). Red: GFAP immunofluorescence; Blue: DAPI staining (nuclei). Scale bar: 50 um.

Figure 5 shows the transversal sections of spinal cord tissues, obtained 5 weeks after SCI from all the experimental groups, that were immunostained using a specific antibody directed against the glial fibrillary acidic protein (GFAP, red) and counterstained using DAPI (blue) as a nuclear dye. Whole SC tissue images were captured in a fluorescence microscope using an objective with 4x magnification. In the sham group micrograph (Figure 5A), an intact and healthy spinal cord tissue with faint GFAP-immunofluorescence (IF) and correct histological distribution of cell nuclei (DAPI) was observed. The mechanical damage produced an important contraction of the tissue section, provoked a clear disruption of cell organization and an intense increase in GFAP-immunoreactivity (IR), as shown in Figure 5B. In the group treated with GnRH (Figure 5C), the tissue was also shrunk, as in the SCI group, but there was a clear disarrangement of the gray and white matter and an increased GFAP-IR, in comparison with the tissue from the sham group. In the tissue collected from animals treated with GH, GFAP-IR was importantly reduced in comparison with the SCI group (Figure 5D), although it appeared to be still higher than the sham control. The effect of the combined treatment (GnRH+GH) apparently showed a similar result as that obtained with the individual treatments, as observed in Figure 5E, in which the tissue was preserved, and the presence of GFAP-IR was decreased in relation to the untreated damaged animals.

**Figure 6:**
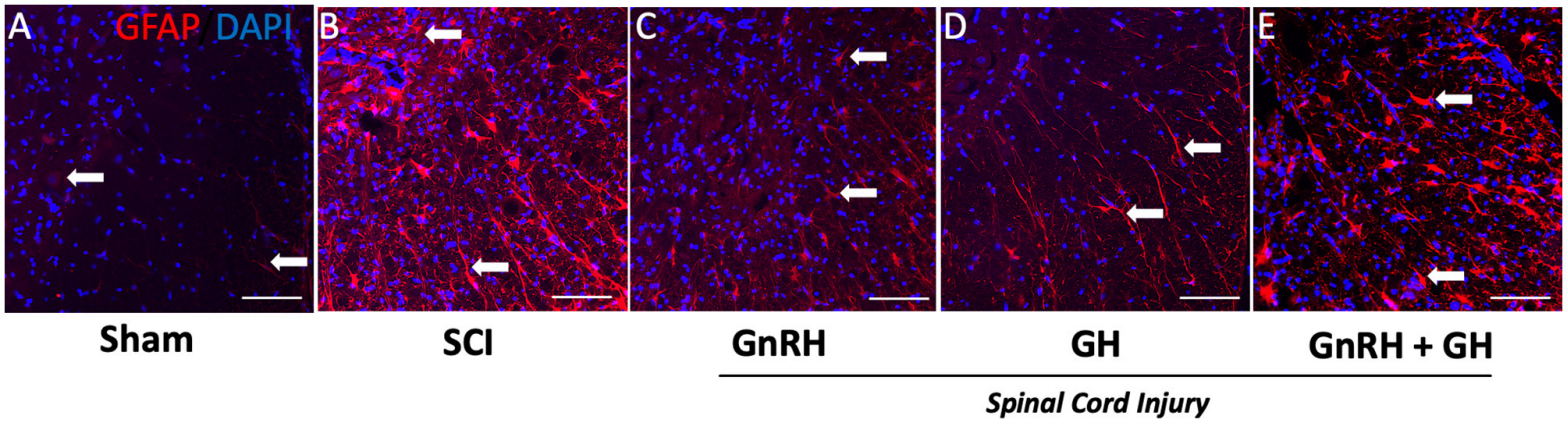
GFAP immunohistochemistry in spinal cord tissue. Transverse sections of the spinal cord collected from the distal side of the injury (T11–T12) at 20x magnification. Experimental groups: **(A)** sham (control), **(B)** spinal cord injury without treatment (SCI), **(C)** GnRH, **(D)** GH, and **(E)** GnRH+GH (G+G). Red: GFAP immunofluorescence; Blue: DAPI staining (nuclei). Arrows show representative immunostaining. Scale bar (50 um).

The effect of the corresponding treatments was better appreciated when the tissues were analyzed at higher magnification (20x), as observed in micrographs of the ventral funiculus (white columns) and ventral horn (gray matter) (Figures 6A–E). As mentioned above, very little GFAP-IR was present in the sham group (Figure 6A); in contrast, an intense IR was found in the SCI-damaged group (Figure 6B). A clear reduction in GFAP-IR was detected in the GnRH (Figure 6C) or GH (Figure 6D) treatments, and to a lesser extent with the combination of both hormones (Figure 6E). The untreated damaged tissue showed spaces in the white matter (arrows); this vacuolation correlated with elongated cell projections with intense GFAP-IR. It was found that both, GnRH and GH, effectively reduced GFAP-IR in comparison with the SCI group. The presence of some remaining astrocyte-like cells (arrows) was also observed, showing signs of cell stress such as elongated fibrillar morphology and intense GFAP immunoreactivity (Figures 6B–D). Intriguingly, cell shape and intensity of GFAP-IR apparently looked similar between the SCI group (Figure 6B) and the animals treated with the combination of GnRH and GH (Figure 6E), suggesting that the co-treatment inhibited the cellular changes induced with the independent administration of each hormone. The increased number of small cell nuclei probably indicates the presence of infiltrated inflammatory cells; interestingly, the treatment with GH showed an apparent decrease in infiltrated cells in comparison with GnRH and the combined treatments (Figure 6D).

### 3.5. Effect of GnRH and GH upon MBP- and Iba1-immunoreactivity after SCI

**Figure 7:**
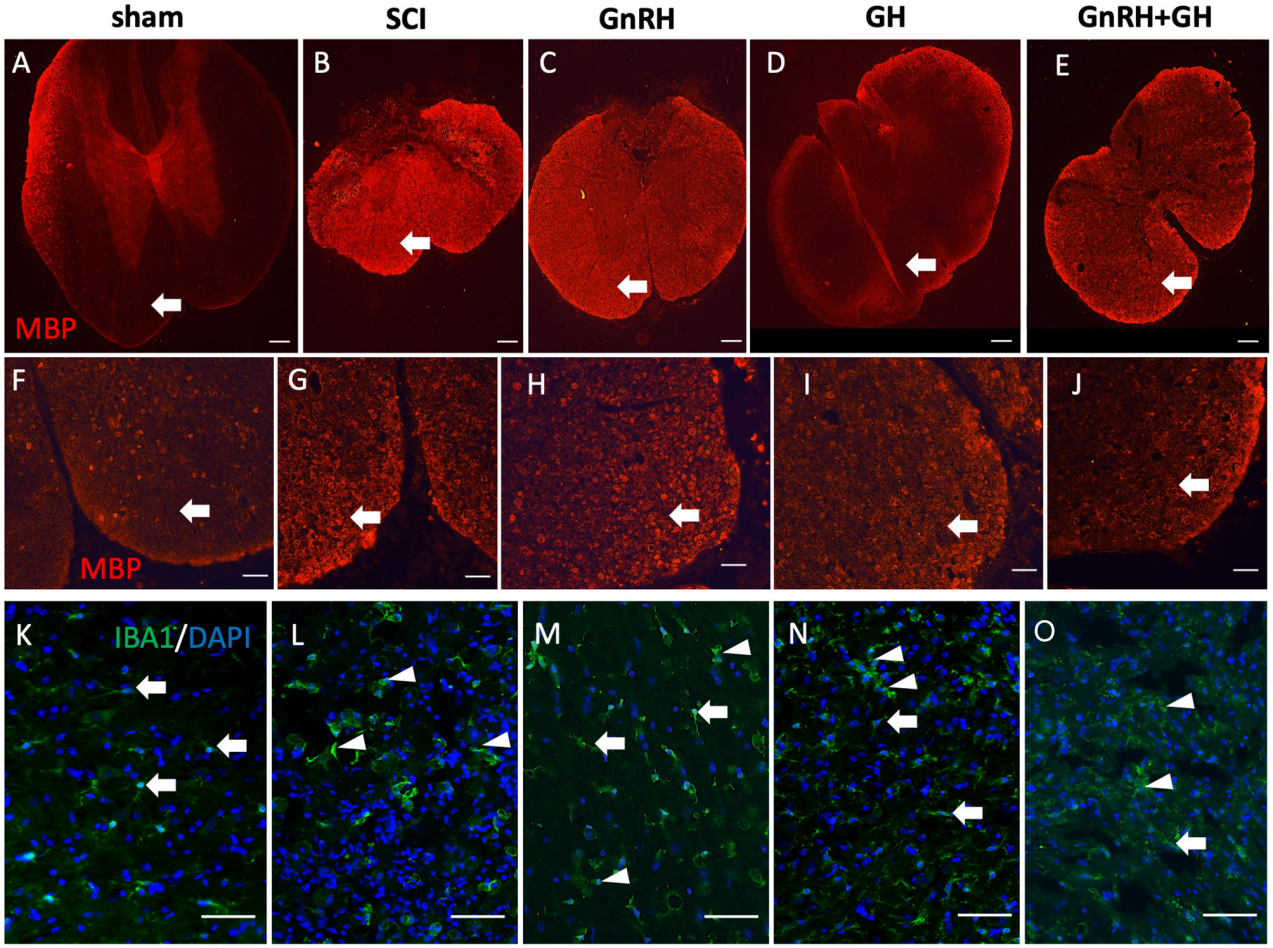
Myelin basic protein (MBP) and Iba1 immunohistochemistry in spinal cord tissue. Transverse sections of the spinal cord collected from the distal side of the injury (T11–T12). MBP immunofluorescence (red) at 4x magnification **(A–E)** and 20x **(F–J)**; Iba1 immunofluorescence (green) at 20x magnification **(K–O)**. Arrows and arrowheads show representative cells (resting and active microglia, respectively). Experimental groups: sham (control), spinal cord injury (SCI), GnRH, GH, and GnRH+GH (G+G). Scale bar [**(A–E)** 50 uM] [**(F–O)** 20 uM].

We also studied how myelin basic protein (MBP) and Iba1 immunoreactivities changed in each condition (Figure 7). As observed in Figures 7A, F, a very low MBP-IR level was located in the ventral white matter of the sham group (arrows); in contrast, a very intense immunofluorescence was present in the tissue obtained from injured animals without treatment (Figures 7B, G). In the case of the GnRH treatment (Figures 7C, H), an intense MBP-IR and proper tissue integrity and organization were found, despite a clearly diminished tissue area in comparison with the sham control. However, the GH-treated group showed that MBP-IR had a similar level than control and the tissue showed histoanatomical integrity (Figures 7D, I), apparently reversing the effects of the damage. The combined treatments protected the tissue from cellular disarrangement but still showed intense MBP-IR in the white matter in comparison with the sham and GH experimental groups (Figures 7E, J).

Iba1 is a well-accepted marker for microglia and infiltrated macrophages in the SNC. Figure 7K shows the presence of microglia-like immunopositive cells (arrows) in the sham group, with classical resting shape and long projections to sense changes in the tissue environment. Interestingly, after SCI, intense immunofluorescent cells with ameboid shape became evident, whereas in the treated tissues a mixture of ameboid and resting shape cells (arrowheads) with many ramifications (arrows; Figures 7K–M) were observed.

### 3.6. Sensorial recovery after SCI in the hot-plate assay

**Figure 8:**
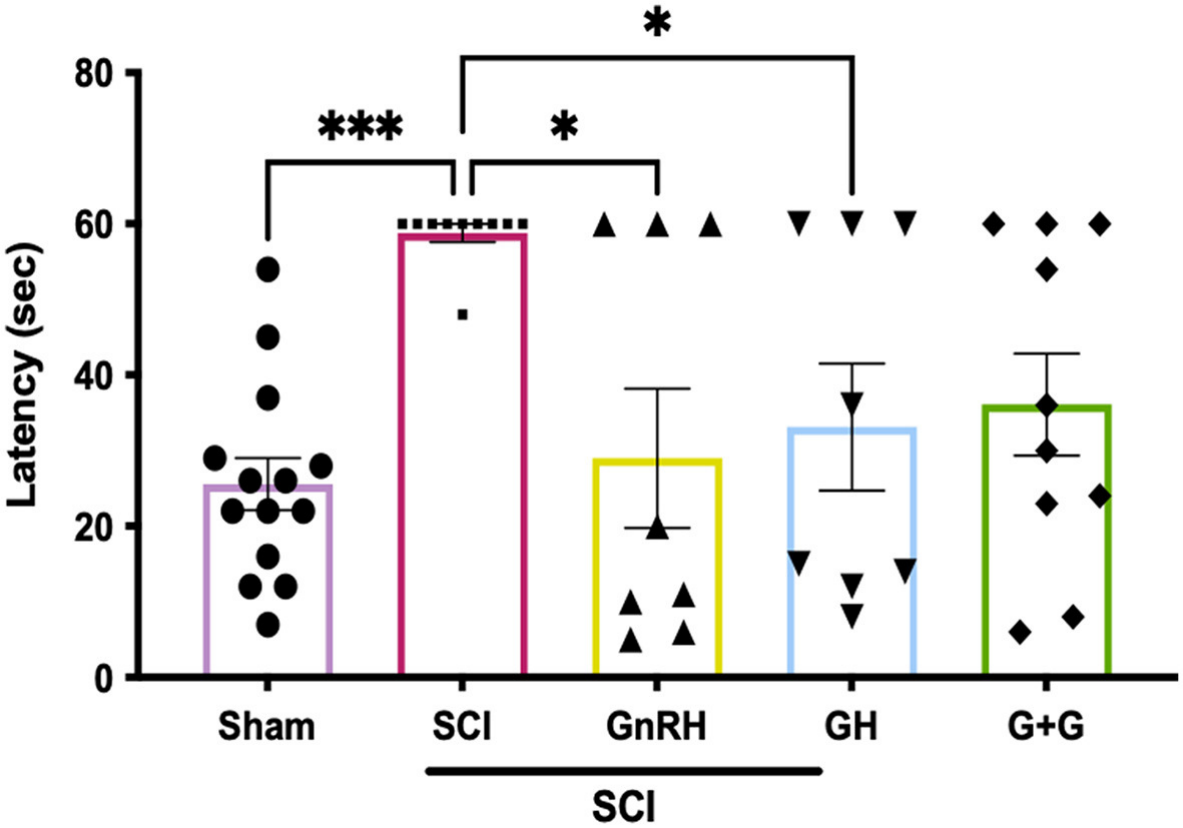
Latency of response in hot-plate assay in animals with SCI; 60 s was set up as maximum time of latency. Experimental groups: sham (control), spinal cord injury (SCI), GnRH, GH, and GnRH+GH (G+G). Units are in seconds (s). Results are shown as mean ± SEM, each dot represents an observation (animal); asterisks *show differences. *p < 0.05; ***p < 0.001 as determined by one-way ANOVA with LSD Fisher as *post hoc* test.

To assess sensorial recovery, hormonal treatments were administered during 5 weeks, following previous reports from our group where a GnRH analog (leuprolide acetate) induced a significant recovery of movement in hand limbs and improved urinary parameters (Yalcin et al., 2009). Here, the latency of response of the animals was recorded in the hot-plate assay (Figure 8); we found a latency of 25.6 s for the sham group, but it increased significantly to 60 s in the injured group (*P* < 0.0001). However, individual treatments induced an important recovery in sensorial function as GnRH reduced latency to 29 s (*p* < 0.05) and GH to 33.1 s (*p* < 0.05) in comparison with the lesioned group, whereas their combination tended to diminish (36.1 s; G+G) but showed a *p*-value of 0.052, without statistical difference.

## 4. Discussion

The chronic administration of GnRH and/or GH significantly reduced the expression of proinflammatory and glial activity markers in the lesioned areas and improved sensory recovery in animals harmed with thoracic SCI. Neural damage was induced mechanically by catheter insufflation at the level of the T10 vertebra, which resulted in an important motor and sensory loss in the hindlimbs; also, the urinary bladder function was impaired, and thus, care support was provided throughout the study. In addition, our objective was to examine the simultaneous activation of canonical receptors for GnRH and GH in a model for neural damage. GHR and GnRHR, both with well-documented neurotrophic capacity and distinct signaling pathways, are widely expressed in the nervous system including the spinal cord (Thörnwall-Le Grevès et al., 2001; Quintanar et al., 2009). Despite the difference in their biochemical structure, and signaling pathway transduction, when activated, both receptors can exert strong neurotrophic actions (Stamatiades and Kaiser, 2018; Chhabra et al., 2021). The proinflammatory microenvironment detected in the spinal cord samples collected from the injury site and surrounding spinal tissue was analyzed through changes in the relative gene expression of interleukin-1β (IL1β), interleukin-6 (IL6), and inducible nitric oxide synthetase (iNOS) (Cheng et al., 2016). Consistently, our determinations in tissues obtained from the caudal side of the injured spinal cord showed a higher anti-inflammatory response to treatments, even in comparison with samples obtained from the injury epicenter. Interestingly, the tissues collected from the cephalic side of the injury did not show an SCI-induced increase in any marker showing a damage progression with caudal orientation. Our gene-expression analysis included tissues collected from the epicenter T9–T10 and the surrounding areas T7–T8 and T11–T12, and we found that the caudal section was the most responsive to both, independent GH or GnRH treatments, and their combination. In that region, all the studied genes showed a consistent decrease in cytokine and glial activity markers demonstrating for the first time an anti-inflammatory and glial-modulatory effect for GH and GnRH in a SCI experimental model. It is documented that right after the primary injury induced by mechanical compression, there is an immediate and enormous local response that includes generalized cell death and a storm of proinflammatory cytokines and chemokines; this initial stage is known as the immediate phase (Anjum et al., 2020). The acute phase goes from 2 to 48 h postinjury and involves a complex dynamic as the activation of local microglia coexists with the infiltration of monocytes and neutrophils, leading to a coordinated response with multiple systems acting at the same time (Freyermuth-Trujillo et al., 2022). Our data suggest that both hormones, GnRH and GH, can modulate the response of microglia, astrocytes, and infiltrated immune cells, including monocytic macrophages through the acute, subacute, and chronic phases, as a critical part of the secondary injury (Gensel and Zhang, 2015; Freyermuth-Trujillo et al., 2022).

During the secondary phase of damage progression, microglia and astrocytes get very active during the acute and subacute stages, in which they proliferate and migrate into the lesion site to produce the glial scar (Ahuja et al., 2017). Glial scarring is an interesting phenomenon that includes well-orchestrated processes looking for healing, but at the same time, they block the axonal regeneration as a product of a physical barrier formed by abnormal extracellular matrix protein accumulation, random cell aggregation, and microambiental imbalance (Fan et al., 2018). There is also an important interaction between infiltrated immune cells, which mainly intercommunicate with microglia and astrocytes to coordinate a physiological response that limits the damage propagation (Bellver-Landete et al., 2019). This study clearly shows an anti-inflammatory effect of GH and GnRH as both can limit and modulate, a SCI-induced increase in the expression of proinflammatory and glial markers. It is known that IL1β, IL6, and inducible iNOS are mainly produced by microglia and macrophages as a result of primary damage, and these proinflammatory mediators get exacerbated during the next 7 to 14 days after injury (Freyermuth-Trujillo et al., 2022). Here, we found that our treatments significantly decreased the expression of IL1β, IL6, and iNOS at day 21 postinjury, suggesting that, at least, the observed effect of GH in the sensory recovery involves anti-inflammatory actions.

The ionized calcium-binding adapter (Iba1) is an accepted molecular marker for microglia/macrophages, an upregulation of its mRNA expression correlates with an increase in the number of resident microglial cells and infiltrated monocytic macrophages in neural damaged tissues (Ohsawa et al., 2004). Our data showed that GnRH and the combined (G+G) treatments reduced the difference in significance of Iba1 expression in relation to the SCI group, suggesting a reduction in the number of microglia/macrophages in the injury site during the acute phase of SCI. Our results could imply a modulatory role of GH and GnRH during the damage-responsive proliferation of microglia and an inhibition of chemotaxic factors that promote macrophage/lymphocyte invasion. Necrotic cell death is a common feature in a traumatic event in the spinal cord, the presence of damage-associated molecular markers triggers a local immediate immune response involving microglial activation followed by the systemic immune response (Shen et al., 2022). Thus, our proposal is that GH and/or GnRH are likely to be acting as early modulators of glial and macrophage response. As expected, the expression of microglial phenotypes M1 (CD86) and M2 (CD206) was significantly increased in untreated tissue collected from the injury site and the distal section. As we hypothesized, CD86 gene expression was downregulated in animals treated with GH and GnRH, suggesting an anti-inflammatory effect. Unexpectedly, M2-microglia phenotype was not increased with the hormonal treatments, and similarly to Iba1 and CD86 expression, we observed a decrease in the CD206 transcription. Recent studies have shown the importance of a healthy activity of microglial cells after a SCI, it is now accepted that exacerbated microglia activity leads to damage progression, whereas, a fine-tuned microglial activity is highly beneficial. Interestingly, the phenotype balance between M1 and M2 microglial types is critical for the healing process as M1 polarization has been associated with a negative prognosis (Kobayashi et al., 2013; Kobashi et al., 2020). The insulin-like factor-1 (IGF-1), a classical mediator for GH actions, has been proved to be produced by the M2-microglia phenotype and its exogenous administration reduced SCI damage (Li et al., 2020a; Nishida et al., 2020). In addition, IGF-1, BDNF, and NT3 are produced in neural tissues and positively respond to GH stimulation (Martínez-Moreno et al., 2016). Complementarily, total ablation of microglia has a detrimental effect as it unleashes an uncontrolled macrophage/lymphocyte infiltration with exacerbated proinflammatory environment (Bellver-Landete et al., 2019). Results obtained by IHC for Iba1 showed a higher number of resting-shape microglia in the tissues obtained from animals treated with GH and GnRH, suggesting an “inactivation” of microglia.

The glial acidic fibrillary protein (GFAP) is a marker for astrocyte reactivity, also known as astrogliosis in spinal cord tissue (Yang and Wang, 2015). Here, we observed that SCI induced changes in cell morphology and showed intense immunofluorescence in relation to the unharmed control group. These observations are consistent with previous reports describing an increase in GFPA-IR and astrocytic fibrillar shape in the damaged SC (Herrmann et al., 2008). In addition, GFAP-immunostaining showed an evident histoanatomical disarrangement induced by SCI and this negative effect in the SC tissue was partially prevented with GH and/or GnRH treatments. However, our qPCR analysis showed the opposite, indicating a reduction in GFAP mRNA expression levels. This could be partially explained by differences in the temporality of the study, as mRNA studies were performed 3 weeks after the injury, whereas the immunohistochemical observations were done 5 weeks after SCI. It is likely that GFAP protein levels remain upregulated for a longer period, which correlates with the fibrillar morphology of the observed astrocytes 5 weeks after SCI. It is important to take mRNA results carefully as changes in transcriptional rate do not always translate immediately into protein synthesis.

Vimentin is also an upregulated marker of reactive gliosis that positively correlates with the magnitude of glial scarring and the inhibition of axonal regeneration in SCI (Xia et al., 2008; Teshigawara et al., 2013). This intermediate filament is one of the main secreted proteins that contribute to astroglial migration and glial scarring. Its inhibition has been shown to result in beneficial effects on functional recovery after neurotrauma (Qian et al., 2015). Here, we found that the expression of vimentin was reduced with growth hormone (GH) and GnRH+GH (G+G) treatments. These results were consistent with the observed changes in GFAP-immunofluorescence (IF), indicating a reduction in reactive gliosis in injured animals. This may be of significance as, for example, in neonatal mice a scar-free healing allows better neural repair (Li et al., 2020b). The data suggest an inhibitory effect of GH and GnRH upon reactive astrogliosis after SCI. An increase in myelin basic protein (MBP) has been reported in spinal cord lesions (Bartholdi and Schwab, 1998), and this effect was also observed in our experiments. GH was able to slightly decrease MBP-IR, but GnRH and the combined treatment showed an increased fluorescence. The immunostaining for MBP allowed to confirm a preventive effect of GH and/or GnRH upon the tissue disorganization, as both white and gray matter showed an improved tissue distribution.

In the injury epicenter section, the combined hormone treatment (G+G) exhibited a significant rise in GH mRNA levels. This was an interesting finding as the pattern that followed the data suggested an additive effect, meaning that neither hormone, independently, exerted an individual action upon this marker. However, we observed that GHR was upregulated only in the epicenter in the GH group in comparison with the sham control; also, the treatment with GH was significantly different in comparison with the SCI group, suggesting that under damage GH is able to upregulate its own receptor suggesting that a possible emergency mechanism gets triggered. This effect was similar to that observed in previous studies in retinal tissue under excitotoxic conditions, which also showed an upregulation of GH receptor mRNA at the injury site (Fleming et al., 2018). Moreover, it is worth noting that GH induced the upregulation of GnRHR in all three sections of tissue analyzed, indicating an increase in GnRH responsivity, but in the G+G, this effect is lost. This finding was particularly intriguing as GH seemed to promote the expression of both receptors, which leads to new research questions on the action mechanisms involved in these effects. As we did not find synergistic neurotrophic responses with the combined treatment, further studies are needed to fully understand their interaction.

Microenvironment imbalance is a drastic dysregulation of cytokines, chemokines, and pro-peptides which figures as the main cause of poor SC tissue regeneration and recovery of SCI (Fan et al., 2018). During this tissue disruption, glial activity gets exacerbated promoting an increase in proinflammatory cytokines and the formation of a glial scar (O'Shea et al., 2017). Here, we found that GnRH and GH may act as homeostatic and survivor factors reducing the inflammatory environment, limiting the glial scar formation which allows the partial recovery of sensory function.

Our data are consistent with previous reports where administration of chronic treatments with GnRH or leuprolide acetate (GnRH analog) resulted in motor function recovery in rats and patients with a similar pattern as observed here in the GnRH group (Calderón-Vallejo and Quintanar, 2012; Calderón-Vallejo et al., 2015). We found a strong correlation between sensory recovery and the downregulation of proinflammatory and glial markers, suggesting that the observed regenerative and/or protective effect of the treatments may involve a controlled microglial response and possibly a reduction in scar formation (currently under research) that allows for functional rehabilitation. It is well accepted that GH can not only induce IGF-1 production in neural tissue but also promote transcription of potent neurotrophic factors, such as BDNF, NT3, BMP4, or GNDF (Martínez-Moreno et al., 2018b; Baltazar-Lara et al., 2021), after neural injury. The observed recovery in sensory function in the spinal cord may involve local synthesis of growth factors, neurotrophins, neurohormones, and peptides, through modulation by GnRH and GH. Although there is no direct evidence about GH in a similar model of SCI, the amount of evidence related to its strong neurotrophic actions has increased substantially. No synergistic effect of GnRH and GH was observed in any marker or tissue section. Interestingly, we found that the spinal cord tissue sampled in the caudal portion of the injury epicenter showed enhanced responsiveness to the treatments, while the tissue collected from the cephalic section responded poorly. These observations led us to conduct histological analysis of the most responsive section, and we found a consistent response in glial activity.

The SCI has a detrimental impact on sensory response as evaluated by the hot-plate test, with a longer latency period observed in lesioned animals that did not receive treatment when compared to those that received GnRH or GH treatment. It is possible that the administration of GnRH and/or GH leads to the survival of neurons in the SCI, similar to what occurs when neurotrophic factors such as BDNF are administered during an inflammatory process (Matayoshi et al., 2005; Ceni et al., 2014). Additionally, GnRH treatment improved the expression of neurofilaments at the lesion site, suggesting axonal growth (Calderón-Vallejo and Quintanar, 2012). Another possibility is that the hormonal treatment induces remyelination, which is supported by the increase in the expression of myelin basic protein (MBP) observed in this study. A previous study also reported that the percentage of spared white matter in the lesion site was greater in GnRH-treated rats compared with untreated animals, and this was correlated with improved functional recovery (Calderón-Vallejo et al., 2015). In addition, our results suggest that the presence of GnRH and GH receptors in neurons, possibly in glial cells, is widely distributed throughout the nervous system and their actions are not limited to their traditional cell targets (Martínez-Moreno et al., 2018a; Martínez-Moreno and Arámburo, 2020). This suggests that at least some of the sensory recovery observed in our work may result from the activation of cells in the damaged or peripheral area. Additionally, it is possible that GnRH and/or GH may act upstream or downstream in the sensorimotor pathway, particularly in the primary cortex, thalamus, and dorsal ganglia. Our treatments may not only serve as anti-inflammatory agents but also protect or restore functional reinnervation through the induction of axogenic and synaptogenic processes.

An interesting observation in this study was that treatment with both hormones, either independently or combined, was associated with a reduction in mortality of the injured animals as well as an improvement in urinary function (unpublished data), as previously reported for LA (Calderón-Vallejo et al., 2015), suggesting that the increase in survival rate in treated rats might be related, at least partially, to urinary function recovery (unpublished data).

In summary, our findings showed that the administration of GnRH or GH can downregulate the expression of cytokine and glial markers at 3 weeks post-damage and subsequently induce sensory recovery at 5 weeks post-SCI. Importantly, we found enhanced responsiveness to the treatments, particularly in the tissue samples collected from the caudal section of the damage epicenter. In this study, we did not observe a synergistic effect of the combined hormonal administration. These results are consistent with emerging literature about the potential clinical use of currently available hormonal treatments to reduce the progression of neural damage after trauma, stroke, hypoxia-ischemia, or even to reduce side effects of neurosurgery.

## Data availability statement

The original contributions presented in the study are included in the article/supplementary material, further inquiries can be directed to the corresponding authors.

## Ethics statement

The animal study was reviewed and approved by Comité de Bioética, Instituto de Neurobiología, Universidad Nacional Autónoma de México - Comité de Bioética, Universidad Autónoma de Aguascalientes.

## Author contributions

Conceptualization: CM-M, DC-V, JQ, and CA. Methodology: DC-V, IH-J, CD-G, JÁ-M, VU-S, JO-H, MC, JB-M, DE, and RB-L. Supervision: CM-M, IH-J, CD-G, and DC-V. Writing—original draft: CM-M, DC-V, JQ, and CA. Writing—review and editing: CA, DC-V, CD-G, JQ, CM-M, ML, and JÁ-M. Resources and funding: JQ, CA, ML, JÁ-M, and CM-M. All authors contributed to the article and approved the submitted version.

## References

[B1] Ahuja C. S. Wilson J. R. Nori S. Kotter M. R. N. Druschel C. Curt A.. (2017). Traumatic spinal cord injury. Nat Rev Dis Primers 3, 17018. 10.1038/nrdp.2017.18 28447605

[B2] Alba-Betancourt C. Luna-Acosta J. L. Ramírez-Martínez C. Ávila-González D. Granados-Ávalos E. Carranza M.. (2013). Neuroprotective effects of growth hormone (GH) after hypoxia-ischemia injury in embryonic chicken cerebellum. Gen. Comp. Endocrinol. 183, 17–31. 10.1016/j.ygcen.2012.12.004 23262274

[B3] Altamira-Camacho M. Medina-Aguiñaga D. Cruz Y. Calderón-Vallejo D. Kovacs K. Rotondo F.. (2020). Leuprolide acetate, a GnRH agonist, improves the neurogenic bowel in ovariectomized rats with spinal cord injury. Dig. Dis. Sci. 65, 423–430. 10.1007/s10620-019-05783-4 31471861

[B4] Anjum A. Yazid M. D. Fauzi Daud M. Idris J. Ng A. M. H. Selvi Naicker A.. (2020). Spinal cord injury: pathophysiology, multimolecular interactions, and underlying recovery mechanisms. Int. J. Mol. Sci. 21, 20. 10.3390/ijms21207533 33066029 PMC7589539

[B5] Arámburo C. Alba-Betancourt C. Luna M. Harvey S. (2014). Expression and function of growth hormone in the nervous system: a brief review. Gen. Comp. Endocrinol. 203, 35–42. 10.1016/j.ygcen.2014.04.035 24837495

[B6] Ávila-Mendoza J. Mora J. Carranza M. Luna M. Arámburo C. (2016). Growth hormone reverses excitotoxic damage induced by kainic acid in the green iguana neuroretina. Gen. Comp. Endocrinol. 234, 57–67. 10.1016/j.ygcen.2016.04.004 27064058

[B7] Baltazar-Lara M. R. Ávila-Mendoza J. Martínez-Moreno C. G. Carranza M. Pech-Pool S. Vázquez-Martínez O.. (2021). Neuroprotective effects of growth hormone (GH) and insulin-like growth factor type 1 (IGF-1) aftr hypoxic-ischemic injury in chicken cerebellar cell cultures. Int. J. Mol. Sci. 22, 256. 10.3390/ijms22010256 33383827 PMC7795313

[B8] Bartholdi D. Schwab M. E. (1998). Oligodendroglial reaction following spinal cord injury in rat: transient upregulation of MBP mRNA. Glia. 23, 278–284. 10.1002/(sici)1098-1136(199807)23:3<278::aid-glia10>3.0.co;2-q 9633812

[B9] Bellver-Landete V. Bretheau F. Mailhot B. Vallieres N. Lessard M. Janelle M. E.. (2019). Microglia are an essential component of the neuroprotective scar that forms after spinal cord injury. Nat. Commun. 10, 518. 10.1038/s41467-019-08446-0 30705270 PMC6355913

[B10] Bianchi V. E. Locatelli V. Rizzi L. (2017). Neurotrophic and neuroregenerative effects of GH/IGF1. Int. J. Mol. Sci. 18, 11. 10.3390/ijms18112441 29149058 PMC5713408

[B11] Brockie S. Hong J. Fehlings M. G. (2021). The role of microglia in modulating neuroinflammation after spinal cord injury. Int. J. Mol. Sci. 22, 18. 10.3390/ijms22189706 34575871 PMC8470129

[B12] Calderón-Vallejo D. Quintanar J. L. (2012). Gonadotropin-releasing hormone treatment improves locomotor activity, urinary function and neurofilament protein expression after spinal cord injury in ovariectomized rats. Neurosci. Lett. 515, 187–190. 10.1016/j.neulet.2012.03.052 22480691

[B13] Calderón-Vallejo D. Quintanar-Stephano A. Hernández-Jasso I. Jiménez-Hernández V. Ruiz-Ornelas J. Jiménez I.. (2015). Functional and structural recovery of the injured spinal cord in rats treated with gonadotropin-releasing hormone. Neurochem. Res. 40, 455–462. 10.1007/s11064-014-1486-9 25618391

[B14] Ceni C. Unsain N. Zeinieh M. P. Barker P. A. (2014). “Neurotrophins in the Regulation of Cellular Survival and Death,” in Neurotrophic Factors. Handbook of Experimental Pharmacology, Lewin, G., and Carter, B. (eds). Berlin, Heidelberg: Springer. 10.1007/978-3-642-45106-5_8 24668474

[B15] Cheng Z. Zhu W. Cao K. Wu F. Li J. Wang G.. (2016). Anti-inflammatory mechanism of neural stem cell transplantation in spinal cord injury. Int. J. Mol. Sci. 17, 9. 10.3390/ijms17091380 27563878 PMC5037660

[B16] Chhabra Y. Lee C. M. M. Muller A. F. Brooks A. J. (2021). GHR signalling: Receptor activation and degradation mechanisms. Mol. Cell. Endocrinol. 520, 111075. 10.1016/j.mce.2020.111075 33181235

[B17] Chu C. Zhou J. Zhao Y. Liu C. Chang P. Zhou Q.. (2013). Expression of FSH and its co-localization with FSH receptor and GnRH receptor in rat cerebellar cortex. J. Mol. Histol. 44, 19–26. 10.1007/s10735-012-9449-4 22972435

[B18] Cuatrecasas G. Kumru H. Coves M. J. Vidal J. (2018). GH deficiency in patients with spinal cord injury: efficacy/safety of GH replacement, a pilot study. Endocr. Connect. 7, 1031–1039. 10.1530/EC-18-0296 30352393 PMC6198193

[B19] Devesa P. Gelabert M. González-Mosquera T. Gallego R. Relova J. L. Devesa J.. (2012). Growth hormone treatment enhances the functional recovery of sciatic nerves after transection and repair. Muscle Nerve. 45, 385–392. 10.1002/mus.22303 22334173

[B20] Díaz Galindo C. Gómez-González B. Salinas E. Calderón-Vallejo D. Hernández-Jasso I. Bautista E.. (2015). Leuprolide acetate induces structural and functional recovery of injured spinal cord in rats. Neural Regen. Res. 10, 1819–1824. 10.4103/1673-5374.170311 26807118 PMC4705795

[B21] Díaz-Galindo C. Calderón-Vallejo D. Hernández-Jasso I. Cervantes-García D. Martínez-Diaz D. Ibarra-Martínez D.. (2021). Gonadotropin-releasing hormone receptor expression in human spinal cord. Neurochem. Res. 46, 165–170. 10.1007/s11064-020-03178-w 33206314

[B22] Díaz-Galindo M. D. C. Calderón-Vallejo D. Olvera-Sandoval C. Quintanar J. L. (2020). Therapeutic approaches of trophic factors in animal models and in patients with spinal cord injury. Growth Fact. 38, 1–15. 10.1080/08977194.2020.1753724 32299267

[B23] Faden A. I. Wu J. Stoica B. A. Loane D. J. (2016). Progressive inflammation-mediated neurodegeneration after traumatic brain or spinal cord injury. Br. J. Pharmacol. 173, 681–691. 10.1111/bph.13179 25939377 PMC4742301

[B24] Fan B. Wei Z. Yao X. Shi G. Cheng X. Zhou X.. (2018). Microenvironment imbalance of spinal cord injury. Cell Transplant. 27, 853–866. 10.1177/0963689718755778 29871522 PMC6050904

[B25] Fleming T. Martínez-Moreno C. G. Carranza M. Luna M. Harvey S. Arámburo C. (2018). Growth hormone promotes synaptogenesis and protects neuroretinal dendrites against kainic acid (KA) induced damage. Gen. Comp. Endocrinol. 265, 111–120. 10.1016/j.ygcen.2018.02.011 29454595

[B26] Freyermuth-Trujillo X. Segura-Uribe J. J. Salgado-Ceballos H. Orozco-Barrios C. E. Coyoy-Salgado A. (2022). Inflammation: a target for treatment in spinal cord injury. Cells. 11, 17. 10.3390/cells11172692 36078099 PMC9454769

[B27] Gensel J. C. Zhang B. (2015). Macrophage activation and its role in repair and pathology after spinal cord injury. Brain Res. 1619, 1–11. 10.1016/j.brainres.2014.12.045 25578260

[B28] Guerout N. (2021). Plasticity of the injured spinal cord. Cells. 10, 8. 10.3390/cells10081886 34440655 PMC8395000

[B29] Harvey S. Hull K. (2003). Neural growth hormone: an update. J. Mol. Neurosci. 20, 1–14. 10.1385/JMN:20:1:1 12663929

[B30] Heredia M. Sánchez-Robledo V. Gómez I. Criado J. M. Fuente A. Devesa J.. (2021). Cell proliferation in the piriform cortex or rats with motor cortex ablation treated with growth hormone and rehabilitation. Int. J. Mol. Sci. 22:5440. 10.3390/ijms22115440 34064044 PMC8196768

[B31] Herrmann J. E. Imura T. Song B. Qi J. Ao Y. Nguyen T. K.. (2008). STAT3 is a critical regulator of astrogliosis and scar formation after spinal cord injury. J. Neurosci. 28, 7231–7243. 10.1523/JNEUROSCI.1709-08.2008 18614693 PMC2583788

[B32] Hu X. Leak R. K. Shi Y. Suenaga J. Gao Y. Zheng P.. (2015). Microglial and macrophage polarization-new prospects for brain repair. Nat. Rev. Neurol. 11, 56–64. 10.1038/nrneurol.2014.207 25385337 PMC4395497

[B33] Juárez-Aguilar E. Olivares-Hernández J. D. Regalado-Santiago C. GarcÍa-GarcÍa F. (2022). The role of growth hormone in hippocampal function. Vitam. Horm. 118, 289–313. 10.1016/bs.vh.2021.11.007 35180930

[B34] Kgosidialwa O. Hakami O. Muhammad Zia-Ul-Hussnain H. Agha A. (2019). Growth hormone deficiency following traumatic brain injury. Int. J. Mol. Sci. 20, 13. 10.3390/ijms20133323 31284550 PMC6651180

[B35] Kobashi S. Terashima T. Katagi M. Nakae Y. Okano J. Suzuki Y.. (2020). Transplantation of M2-deviated microglia promotes recovery of motor function after spinal cord injury in mice. Mol. Ther. 28, 254–265. 10.1016/j.ymthe.2019.09.004 31604678 PMC6952178

[B36] Kobayashi K. Imagama S. Ohgomori T. Hirano K. Uchimura K. Sakamoto K.. (2013). Minocycline selectively inhibits M1 polarization of microglia. Cell Death Dis. 4, e525. 10.1038/cddis.2013.54 23470532 PMC3613832

[B37] Kroner A. Rosas Almanza J. (2019). Role of microglia in spinal cord injury. Neurosci. Lett. 709, 134370. 10.1016/j.neulet.2019.134370 31283964

[B38] Li H. Kong R. Wan B. Yang L. Zhang S. Cao X.. (2020a). Initiation of PI3K/AKT pathway by IGF-1 decreases spinal cord injury-induced endothelial apoptosis and microvascular damage. Life Sci. 263, 118572. 10.1016/j.lfs.2020.118572 33065147

[B39] Li Y. He X. Kawaguchi R. Zhang Y. Wang Q. Monavarfeshani A.. (2020b). Microglia-organized scar-free spinal cord repair in neonatal mice. Nature. 587, 613–618. 10.1038/s41586-020-2795-6 33029008 PMC7704837

[B40] Martínez-Moreno C. G. Arámburo C. (2020). Growth hormone (GH) and synaptogenesis. Vitam. Horm. 114, 91–123. 10.1016/bs.vh.2020.04.001 32723552

[B41] Martínez-Moreno C. G. Ávila-Mendoza J. Wu Y. Arellanes-Licea Edel C. Louie M. Luna M.. (2016). Neuroprotection by GH against excitotoxic-induced cell death in retinal ganglion cells. Gen. Comp. Endocrinol. 234, 68–80. 10.1016/j.ygcen.2016.03.023 27129619

[B42] Martínez-Moreno C. G. Calderón-Vallejo D. Harvey S. Arámburo C. Quintanar J. L. (2018a). Growth hormone (GH) and gonadotropin-releasing hormone (gnrh) in the central nervous system: a potential neurological combinatory therapy? *Int. J. Mol. Sci*. 19(2). 10.3390/ijms19020375 29373545 PMC5855597

[B43] Martínez-Moreno C. G. Epardo D. Balderas-Márquez J. E. Fleming T. Carranza M. Luna M.. (2019). Regenerative effect of growth hormone (GH) in the retina after kainic acid excitotoxic damage. Int. J. Mol. Sci. 20(18). 10.3390/ijms20184433 31509934 PMC6770150

[B44] Martínez-Moreno C. G. Fleming T. Carranza M. Ávila-Mendoza J. Luna M. Harvey S.. (2018b). Growth hormone protects against kainate excitotoxicity and induces BDNF and NT3 expression in chicken neuroretinal cells. Exp. Eye Res. 166, 1–12. 10.1016/j.exer.2017.10.005 29030174

[B45] Matayoshi S. Jiang N. Katafuchi T. Koga K. Furue H. Yasaka T.. (2005). Actions of brain-derived neurotrophic factor on spinal nociceptive transmission during inflammation in the rat. J. Physiol. 569, 685–95. 10.1113/jphysiol.2005.095331 16210356 PMC1464224

[B46] Meazza C. Pagani S. Travaglino P. Bozzola M. (2004). Effect of growth hormone (GH) on the immune system. Pediatr. Endocrinol. Rev. 1 Suppl 3, 490–495. 16444180

[B47] Millar R. P. (2005). GnRHs and GnRH receptors. Anim. Reprod. Sci. 88, 5–28. 10.1016/j.anireprosci.2005.05.032 16140177

[B48] Nishida F. Zanuzzi C. N. Sisti M. S. Falomir Lockhart E. Camiña A. E. Hereñú C. B.. (2020). Intracisternal IGF-1 gene therapy abrogates kainic acid-induced excitotoxic damage of the rat spinal cord. Eur. J. Neurosci. 52, 3339–3352. 10.1111/ejn.14876 32573850

[B49] Ohsawa K. Imai Y. Sasaki Y. Kohsaka S. (2004). Microglia/macrophage-specific protein Iba1 binds to fimbrin and enhances its actin-bundling activity. J. Neurochem. 88, 844–856. 10.1046/j.1471-4159.2003.02213.x 14756805

[B50] Okada S. Hara M. Kobayakawa K. Matsumoto Y. Nakashima Y. (2018). Astrocyte reactivity and astrogliosis after spinal cord injury. Neurosci. Res. 126, 39–43. 10.1016/j.neures.2017.10.004 29054466

[B51] Olivares-Hernández J. D. Balderas-Márquez J. E. Carranza M. Luna M. Martínez-Moreno C. G. Arámburo C. (2021). Growth hormone (GH) enhances endogenous mechanisms of neuroprotection and neuroplasticity after oxygen and glucose deprivation injury (OGD) and reoxygenation (OGD/R) in chicken hippocampal cell cultures. Neural Plast. 2021, 9990166. 10.1155/2021/9990166 34567109 PMC8461227

[B52] Olivares-Hernández J. D. Carranza M. Balderas-Márquez J. E. Epardo D. Baltazar-Lara R. Ávila-Mendoza J.. (2022). Neuroprotective and regenerative effects of growth hormone (GH) in the embryonic chicken cerebral pallium exposed to hypoxic-ischemic (HI) injury. Int. J. Mol. Sci. 23(16). 10.3390/ijms23169054 36012320 PMC9409292

[B53] O'Shea T. M. Burda J. E. Sofroniew M. V. (2017). Cell biology of spinal cord injury and repair. J. Clin. Invest. 127, 3259–3270. 10.1172/JCI90608 28737515 PMC5669582

[B54] Qian B. J. You L. Shang F. F. Liu J. Dai P. Lin N.. (2015). Vimentin regulates neuroplasticity in transected spinal cord rats associated with micRNA138. Mol. Neurobiol. 51, 437–447. 10.1007/s12035-014-8745-2 24874717

[B55] Quintanar J. L. Diaz-Galindo C. Calderón-Vallejo D. Hernández-Jasso I. Rojas F. Medina-Aguinaga D.. (2018). Neurological improvement in patients with chronic spinal cord injury treated with leuprolide acetate, an agonist of GnRH. Acta Neurobiol Exp (Wars) 78, 352–357. 10.21307/ane-2018-034 30624434

[B56] Quintanar J. L. Salinas E. Gonzalez R. (2009). Gonadotropin-releasing hormone receptor in spinal cord neurons of embryos and adult rats. Neurosci. Lett. 461, 21–24. 10.1016/j.neulet.2009.06.028 19539704

[B57] Sanchez-Bezanilla S. Âberg N. D. Crock P. Walker F. R. Nilsson M. Isgaard J.. (2020a). Growth hormone promotes motor function after experimental stroke and enhances recovery-promoting mechanisms within the peri-infarct area. Int. J. Mol. Sci. 21, 606. 10.3390/ijms21020606 31963456 PMC7013985

[B58] Sanchez-Bezanilla S. Âberg N. D. Crock P. Walker F. R. Nilsson M. Isgaard J.. (2020b). Growth hormone treatment promotes remote hippocampal plasticity after experimental cortical stroke. Int. J. Mol. Sci. 21, 4563. 10.3390/ijms21124563 32604953 PMC7349868

[B59] Shen H. Xu B. Yang C. Xue W. You Z. Wu X.. (2022). A DAMP-scavenging, IL-10-releasing hydrogel promotes neural regeneration and motor function recovery after spinal cord injury. Biomaterials. 280, 121279. 10.1016/j.biomaterials.2021.121279 34847433

[B60] Soendergaard C. Young J. A. Kopchick J. J. (2017). Growth hormone resistance-special focus on inflammatory bowel disease. Int. J. Mol. Sci. 18(5). 10.3390/ijms18051019 28486400 PMC5454932

[B61] Spaziani M. Tarantino C. Tahani N. Gianfrilli D. Sbardella E. Lenzi A.. (2021). Hypothalamo-pituitary axis and puberty. Mol. Cell. Endocrinol. 520, 111094. 10.1016/j.mce.2020.111094 33271219

[B62] Stamatiades G. A. Kaiser U. B. (2018). Gonadotropin regulation by pulsatile GnRH: Signaling and gene expression. Mol. Cell. Endocrinol. 463, 131–141. 10.1016/j.mce.2017.10.015 29102564 PMC5812824

[B63] Teshigawara K. Kuboyama T. Shigyo M. Nagata A. Sugimoto K. Matsuya Y.. (2013). A novel compound, denosomin, ameliorates spinal cord injury via axonal growth associated with astrocyte-secreted vimentin. Br. J. Pharmacol. 168, 903–919. 10.1111/j.1476-5381.2012.02211.x 22978525 PMC3631379

[B64] Thörnwall-Le Grevès M. Zhou Q. Lagerholm S. Huang W. Le Greves P. Nyberg F. (2001). Morphine decreases the levels of the gene transcripts of growth hormone receptor and growth hormone binding protein in the male rat hippocampus and spinal cord. Neurosci. Lett. 304, 69–72. 10.1016/S0304-3940(01)01757-8 11335057

[B65] Vanicky I. Urdzikova L. Saganova K. Cizkova D. Galik J. (2001). A simple and reproducible model of spinal cord injury induced by epidural balloon inflation in the rat. J. Neurotrauma 18, 1399–1407. 10.1089/08977150152725687 11780869

[B66] Xia Y. Zhao T. Li J. Li L. Hu R. Hu S.. (2008). Antisense vimentin cDNA combined with chondroitinase ABC reduces glial scar and cystic cavity formation following spinal cord injury in rats. Biochem. Biophys. Res. Commun. 377, 562–566. 10.1016/j.bbrc.2008.10.024 18930033

[B67] Yalcin I. Charlet A. Freund-Mercier M. J. Barrot M. Poisbeau P. (2009). Differentiating thermal allodynia and hyperalgesia using dynamic hot and cold plate in rodents. J. Pain. 10, 767–773. 10.1016/j.jpain.2009.01.325 19409860

[B68] Yang Z. Wang K. K. (2015). Glial fibrillary acidic protein: from intermediate filament assembly and gliosis to neurobiomarker. Trends Neurosci. 38, 364–374. 10.1016/j.tins.2015.04.003 25975510 PMC4559283

